# Disparity between Post-thaw Survival and Functional Integrity of Vitrified-thawed Oocytes from Obese Mice

**DOI:** 10.5935/1518-0557.20260020

**Published:** 2026

**Authors:** Sandhya Kumari, Aparna Chelvi, Vanishree Vasave Madhvacharya, Aishwarya Ashok, Mouna Bannur Karunakara, Yashas Kiran Ninjoor, Jyolsna Ponnaratta Kunhiraman, Amrutha Nedumbrakkad Kunnath, Keerthana Karunakar Poojary, Sneha Guruprasad Kalthur, Satish Kumar Adiga, Guruprasad Kalthur

**Affiliations:** 1 Division of Reproductive Biology, Department of Reproductive Science, Kasturba Medical College, Manipal Academy of Higher Education, Manipal 576104, Karnataka, India; 2 Centre of Excellence in Clinical Embryology, Department of Reproductive Science, Kasturba Medical College, Manipal Academy of Higher Education, Manipal 576104, Karnataka, India; 3 Department of Anatomy, Kasturba Medical College, Manipal Academy of Higher Education, Manipal 576104, Karnataka, India; 4 Current affiliation: Department of Anatomy, Sree Mookambika Institute of Medical Sciences, Padanilam, Kulasekharam 629161, Tamil Nadu, India; 5 Current affiliation: Sree Mookambika Centre for Research and Innovation, Sree Mookambika Institutes, Padanilam, Kulasekharam 629161, Tamil Nadu, India

**Keywords:** high-fat diet, cryotolerance, spindle defects, oxidative stress, ER stress

## Abstract

**Objective:**

Obesity in women is known to be associated with compromised ovarian function. The oocytes are characterized by elevated cytoplasmic oxidative stress and poor cytoplasmic organization under such conditions, which might affect their tolerance to exogenous stresses such as the freeze-thaw process. This study investigates the cryosusceptibility of oocytes derived from mice with high-fat diet (HFD)-induced obesity.

**Methods:**

Three-weeks-old female mice were divided into a control group (fed a normal chow diet) and an obese group (fed with HFD for 12 weeks). Oocytes from both groups were subjected to vitrification and thawing. Post-thaw survival, reactive oxygen species (ROS) levels, mitochondrial and endoplasmic reticulum (ER) distribution, ER stress, and the maturation potential of GV stage oocytes were evaluated.

**Results:**

Despite increased lipid accumulation and higher ROS levels observed in the oocytes of obese mice, the post-thaw survival rate of GV oocytes was comparable between the groups. However, obesity induced alterations in mitochondrial and ER distribution. Additionally, MII oocytes derived from vitrified-thawed germinal vesicle (GV) stage oocytes of obese mice showed a significantly higher percentage of spindle defects. A notable increase in ER stress markers GRP78 and ATF4 was detected in frozen-thawed GV oocytes compared to the control group.

**Conclusions:**

These findings suggest that maternal obesity does not significantly affect the cryosurvival of oocytes, but it does compromise oocyte quality after vitrification, underscoring the need for optimized cryopreservation strategies in obese patients undergoing ART.

## INTRODUCTION

Obesity is a major health issue worldwide and is linked to infertility. The incidence of obesity has tripled since 1975. The global prevalence of obesity is higher in women than in men. It is predicted that by 2030, over 1 billion people globally will be living with obesity, including 20% of women, highlighting a greater burden of obesity among women (World Obesity Atlas, 2022). A recent survey conducted in India has shown a similar trend, where more than 135 million individuals were obese, with a higher prevalence in women (Ahirwar & Mondal, 2019; Chaudhary *et al*., 2023).

Obesity has been proven to have harmful effects on reproductive health, especially in women, as it is associated with endocrine disorders (Chaudhary *et al*., 2023; Wei *et al*., 2022; Zain & Norman, 2008). It is associated with poor ovarian function and changes in the follicular microenvironment due to increased oxidative stress (Snider & Wood, 2019) and elevated levels of inflammatory markers (Broughton & Moley, 2017). Research shows that a high body mass index (BMI) negatively impacts oocyte and embryo quality (Dağ & Dilbaz, 2015; Hou *et al*., 2016; Lashen *et al*., 2004; Robker, 2008; Yang *et al*., 2012). Lipotoxicity, a common metabolic disturbance in the oocytes of obese women, results from increased free fatty acids, leading to elevated reactive oxygen species (ROS), heightened endoplasmic reticulum (ER) stress (Alves *et al*., 2015), impaired mitochondrial membrane potential, a higher risk of aneuploidy (Muhammad *et al*., 2023), more miscarriages (Correa & Marcinkevage, 2013), fetal malformations (Malasevskaia *et al*., 2021), and poor outcomes in assisted reproductive technology (ART) (Rao *et al*., 2020; Sermondade *et al*., 2019; Supramaniam *et al*., 2018). In our earlier study, we reported that oocytes from obese mice have increased intracellular lipids, higher oxidative stress, and greater ER stress (Rao *et al*., 2020). The compromised cytoplasmic quality and related functional changes in oocytes might affect their cryosurvival. To explore these aspects, the present study aimed to assess whether maternal obesity influences cryosurvival and the post-thaw developmental competence of oocytes under *in vitro* conditions.

## MATERIALS AND METHODS

### Animals and dietary intervention

**Figure 1:**
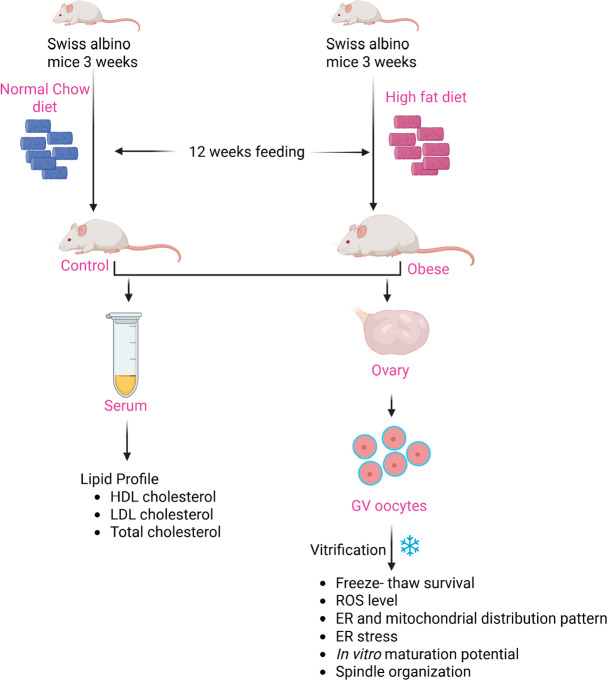
Schematic diagram of the experimental design

Inbred female Swiss albino mice (3 weeks old) with an average weight of 15±1 g, maintained at the Central Animal Research Facility, Kasturba Medical College, Manipal, part of the Manipal Academy of Higher Education, Manipal, were used for the experiments. All experiments and animal handling were conducted according to the institutional guidelines and the national guidelines set by the Committee for the Control and Supervision of Experiments on Animals (CPCSEA). Prior approval was obtained from the Institutional Animal Ethical Committee (IAEC/KMC/105/2019, IAEC/KMC/137/2020, and IAEC/KMC/83/2023), and animal studies were carried out in accordance with ARRIVE guidelines. Animals were housed in a continuously controlled environment, with standard conditions of temperature (25±2°C), humidity (45-55%), and a 12-hours light/12-hours dark cycle, with food and water available *ad libitum*. Mice were randomly divided into a control group (n=41), fed with a standard chow diet, and an obese group (n=43), fed a high-fat diet (HFD; Star Enterprise, Mumbai, India) for 12 weeks (Figure 1) (Poojary *et al*., 2022).

### Serum lipid profile

After 12 weeks of dietary intervention, the mice from control and obese groups were humanely sacrificed. Blood was collected from the heart, and serum was separated. Levels of total cholesterol (Cat. No. 11403002), high-density lipoprotein cholesterol (Cat. No. 11414003), and low-density lipoprotein cholesterol (Cat. No. 11415003) were measured using specific kits from Agappe Diagnostics, India, as described earlier (Rao *et al*., 2020).

### Collection of germinal vesicle (GV) stage oocytes and *in vitro* maturation (IVM)

Ovaries from control (n=21) and obese (n=28) mice were teased in Dulbecco’s Modified Eagle’s Medium (DMEM) culture medium to collect GV stage oocytes. The oocytes were randomly divided into fresh and vitrified-thawed (VT) groups. In the VT group, oocytes underwent vitrification and subsequent thawing. The survival rate was recorded 3 hours after thawing. The fresh or VT group oocytes were either fixed for further analysis or used for *IVM*. For IVM, both fresh and vitrified-thawed GV oocytes from control and obese groups were incubated in IVM medium for 24 hours at 37°C, 5% CO_2_. After 24 hours, the oocytes were assessed for nuclear maturity, and the maturation rate was calculated (Rao *et al*., 2020).

### Vitrification and thawing of oocytes

The vitrification and thawing of oocytes were performed as previously described by Cha *et al*. (2011) with minor modifications. All solutions used for vitrification and thawing were prepared in M2 media containing 20% BSA (Cat. No. A3311, Sigma Aldrich, USA). The equilibrium solution (ES) consisted of 7.5% (v/v) dimethyl sulfoxide (DMSO) and 7.5% ethylene glycol (v/v). The vitrification solution (VS) contained 15% DMSO (v/v) and 15% ethylene glycol (v/v). The thawing solution (TS) contained 1 M sucrose. Dilution solution 1 (DS1) contained 0.5 M sucrose, and dilution solution 2 (DS2) contained 0.25 M sucrose. GV stage oocytes were first transferred to the ES and then to the VS for less than 1 minute. The oocytes were loaded onto the cryolock (CRYOLOCK, Biotech, Inc., USA) with less than 1 µL of VS and immediately plunged into liquid nitrogen. A maximum of five oocytes were loaded onto each cryolock. For thawing, oocytes were removed from liquid nitrogen and immersed in prewarmed TS for less than 1 minute. They were then incubated in DS1 for 3-5 minutes, followed by incubation in DS2 for 3-5 minutes at room temperature. After thawing, the oocytes were washed in a washing solution (WS) containing M2 media with 20% BSA and further incubated in media for up to 3 hours to assess post-thaw survival. These oocytes were used to evaluate various parameters.

### Lipid droplet accumulation by Nile red staining

Nile red staining was performed on 4% paraformaldehyde (PFA)-fixed GV oocytes to assess intracellular lipid levels (Yang *et al*., 2010). Briefly, the GV oocytes were placed in 10 µg/mL Nile red (Cat. No. 72485, Sigma Aldrich, USA) for 10 minutes, counterstained with DAPI (4’,6-diamidino-2-phenylindole, Cat. No. D9542, Sigma Aldrich, USA), and mounted on a glass slide. The stained oocytes were observed under a fluorescence microscope and imaged (Axio Imager A1, Carl Zeiss, Göttingen, Germany). Fluorescence intensity, which indicates lipid levels, was measured using Q-capture software (Q-Capture Pro 7, USA).

### Assessment of intracellular reactive oxygen species (ROS) level in GV oocytes

The ROS levels in GV stage oocytes were measured using the 2′,7′-Dichlorofluorescein diacetate (DCFH-DA) assay as described by Kalthur *et al*. (2016). Briefly, GV stage oocytes were incubated in pre-warmed DMEM droplets containing 10 µM DCFH-DA for 30 minutes at 37°C in 5% CO_2_. After incubation, the oocytes were thoroughly rinsed in PBS containing 0.1% bovine serum albumin (BSA) and mounted on a slide. The oocytes were then examined under a fluorescence microscope, and fluorescence intensity was measured using Q-capture software.

### Distribution pattern of ER and Mitochondria

Oocytes were evaluated for the distribution pattern of ER and mitochondria using ER-Tracker and Mito-Tracker dyes, respectively (Kalthur *et al*., 2016). Stock solutions (1 mM) of ER-Tracker (ER-tracker^TM^ Red, Cat. No. E34250, ThermoFisher Scientific, USA) or Mito-Tracker (Mito-Tracker^TM^ Green FM, Cat. No. M7514, ThermoFisher Scientific, USA) were prepared in DMSO. The GV stage oocytes were incubated in a 20 µL droplet of DMEM media containing 10 µM of either ER-Tracker or Mito-Tracker dyes, overlaid with mineral oil in a Petri dish, and incubated for 20 minutes at 37°C with 5% CO_2_. After incubation, the oocytes were washed in DMEM media, mounted on a glass slide using DPX, and observed under a fluorescence microscope. The percentage of oocytes with a uniform or aggregated distribution pattern of ER and mitochondria was then calculated.

### Spindle organization

*In vitro* matured MII oocytes were washed in PBS and incubated in extraction buffer for 40-60 minutes at 37°C before being fixed in ice-cold methanol for 12 minutes. After fixation and PBS washing, the oocytes were transferred to blocking solution and incubated for 1 hour at 37°C. They were then incubated overnight at 4°C with a monoclonal anti-α-tubulin antibody (Cat. 1:500 dilution. No. T9028, Sigma Aldrich, USA). Subsequently, the oocytes were incubated at 37°C for 1 hour with FITC-conjugated goat anti-mouse IgG antibody (Cat. 1:500 dilution. No. sc-2010, Santa Cruz Biotechnology, USA). After incubation, the oocytes were washed in PBS with 0.1% BSA and counterstained with DAPI. The oocytes were observed under a fluorescence microscope, and the spindle structures were classified as normal or abnormal based on chromosomal alignment and spindle organization (Rao *et al*., 2020).

### Assessing ER stress by immunofluorescence

The PFA-fixed GV stage oocytes were washed, and permeabilization was performed for 15 minutes. The oocytes were then incubated with blocking buffer for 1 hour, followed by an overnight incubation at 4°C with primary antibodies (1:300 anti-GRP78, Cat. No. SAB4501452, Sigma Aldrich, USA; 1:250 anti-ATF4, Cat. No. PA5-68802, Invitrogen, USA; 1:500 anti-ATF6, Cat. No. PA5-20215, Invitrogen; and 1:200 dilution anti-XBP1, Cat. No. ab37152, Abcam, UK). After washing with PBS, they were incubated with anti-rabbit IgG Alexa Fluor 488 (Cat. No. ab150077, Abcam, UK) for 1 hour at 37°C. The oocytes were counterstained with DAPI, observed under a fluorescent microscope, and the expression was quantified based on signal intensity using Q-capture software.

### Statistical analysis

The statistical analysis was performed using two-way ANOVA (ordinary) and an unpaired two-tailed t-test with GraphPad Prism 10.4.1 (GraphPad Inc., USA). Qualitative data (post-thaw survival rate, maturation rate) were compared using Fisher’s exact test (two-sided). Differences were considered significant when *p*<0.05, and data were expressed as the mean ± standard error of mean (SEM).

## RESULTS

### Effect of HFD feeding on body weight and serum lipid profile

The mice fed with HFD for 12 weeks showed a significant gain in body weight (*p*<0.001), as well as increases in serum total cholesterol (*p*<0.01), high-density lipoprotein cholesterol (*p*<0.01), and low-density lipoprotein cholesterol (*p*<0.001) compared to the control mice (Table 1).

**Table 1:** Impact of a high-fat diet (HFD) on body weight, and serum lipid profile in adult Swiss albino mice after 12 weeks of feeding.

Parameter	Groups		*Significance*(p-value)
	Control	Obese	
Gain in body weight (g)	14.84±0.89 (n=41)	20.38±0.89 (n=43)	*p*<0.001
HDL cholesterol (mg/dL)	434.30±32.50 (n=11)	608.50±43.24 (n=9)	*p*<0.01
LDL cholesterol (mg/dL)	18.84±1.45 (n=11)	52.08±3.01 (n=9)	*p*<0.001
Total cholesterol (mg/dL)	58.63±5.22 (n=11)	93.00±9.03 (n=9)	*p*<0.01

Data are presented as mean ± SEM. HDL - high-density lipoprotein; LDL - low-density lipoprotein. “n” represents the number of animals used.

### Effect of HFD-induced obesity on intracellular lipid accumulation, post-thaw survival, and maturation potential of GV oocytes during vitrification

**Figure 2:**
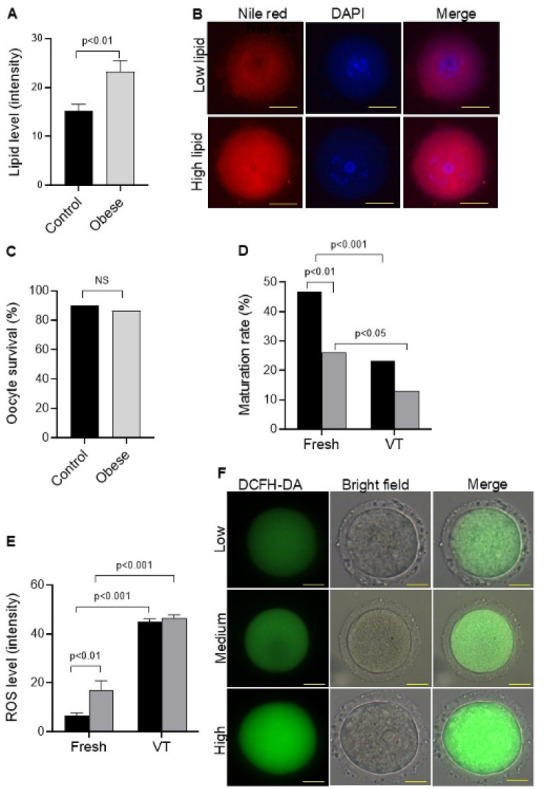
Effect of high-fat diet-induced obesity on **(A)** lipid droplet accumulation in oocytes from control (n=27) and obese group (n=29), assessed by Nile red staining; **(B)** representative images showing low and high lipid accumulation in GV oocytes (400x magnification); **(C)** survival of GV oocytes after vitrification and thawing (control: n=130; obese: n=101); **(D)** nuclear maturation potential in GV oocytes after vitrification and thawing (fresh control: n=79, obese: n=50; vitrified control: n=35, obese: n=42); **(E)** intracellular reactive oxygen species (ROS) levels in GV oocytes (fresh control: n=11, obese: n=8; vitrified control: n=11, obese: n=11); **(F)** representative images showing intracellular ROS levels in GV oocytes stained with 2,7-dichlorodihydrofluorescein diacetate (400x magnification). The scale bar equals 20 µm. Data are presented as mean ± SEM. Control: 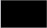 Obese: 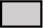

The oocytes from obese mice showed a significantly higher level of intracellular lipid accumulation (*p*<0.01) compared to the oocytes from control mice (Figure 2, A and B). GV oocytes from control and obese mice exhibited similar post-thaw survival rates (Figure 2C). Furthermore, GV oocytes derived from obese mice had a significantly lower (*p*<0.01) maturation rate compared to control group oocytes (Figure 2D). Vitrification and thawing significantly reduced the maturation rate in both control (23%, *p*<0.001) and obese (13%, *p*<0.05) groups compared to the maturation potential of the fresh GV oocytes from each respective group.

### ROS levels in the GV oocytes

Diet-induced obesity significantly increased oxidative stress (*p*<0.01) in GV stage oocytes compared to controls (Figure 2, E and F). Vitrification-thawing caused a notable rise in oxidative stress (*p*<0.001) in oocytes from both the control group (44.96±1.19) and obese group (46.62±1.18) compared to their respective fresh oocytes. However, the vitrification-thawing process did not significantly alter ROS levels between control and obese group oocytes.

### Effect on the distribution pattern of ER and mitochondria

**Figure 3:**
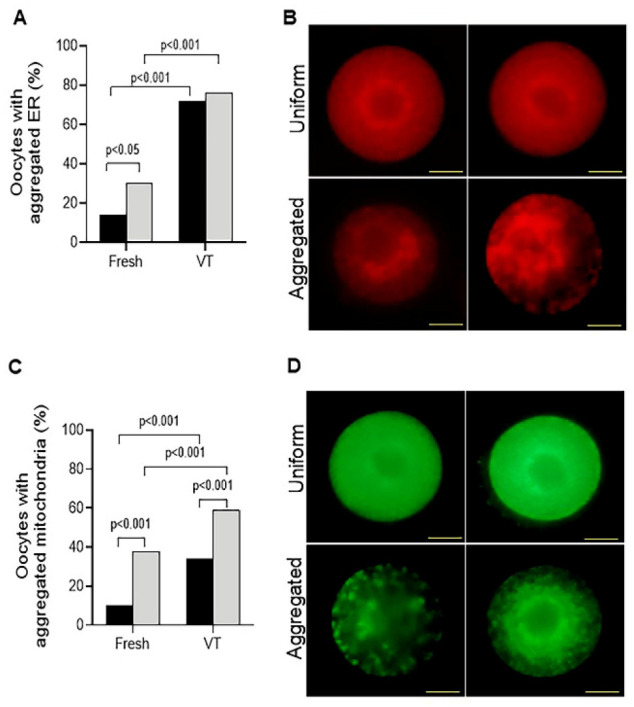
Effect of high-fat diet on organelle distribution patterns in GV oocytes from fresh and vitrified-thawed samples. Percentage of GV oocytes (n=23 oocytes/group) showing an aggregated distribution pattern of **(A)** ER; **(B)** and representative images of different ER distribution patterns in oocytes stained with ER-tracker (400x magnification); **(C)** mitochondrial distribution pattern; **(D)** representative images of different mitochondrial distribution patterns in oocytes stained with Mito-tracker (400x magnification). The scale bar indicates 20 µm. Control: 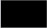 Obese: 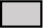

A significant increase (*p*<0.05) in the percentage of oocytes with aggregated ER was observed in the obese group compared to the control (Figure 3, A and B). The percentage of oocytes with aggregated ER increased further significantly (*p*<0.001) after vitrification in both control and obese groups. Similarly, there were significant differences in mitochondrial distribution patterns between the groups. A significantly higher (p<0.001) percentage of oocytes showed aggregated mitochondria in the obese group compared to the control group (Figure 3, C and D). After vitrification and thawing, the proportion of oocytes with aggregated mitochondrial distribution significantly increased (*p*<0.001) in both control and obese groups. Moreover, vitrified-thawed oocytes from the obese group showed a significantly (*p*<0.001) higher percentage of aggregated mitochondria than those from the vitrified-thawed control group.

### Effect on meiotic spindle organization in IVM oocytes

**Figure 4:**
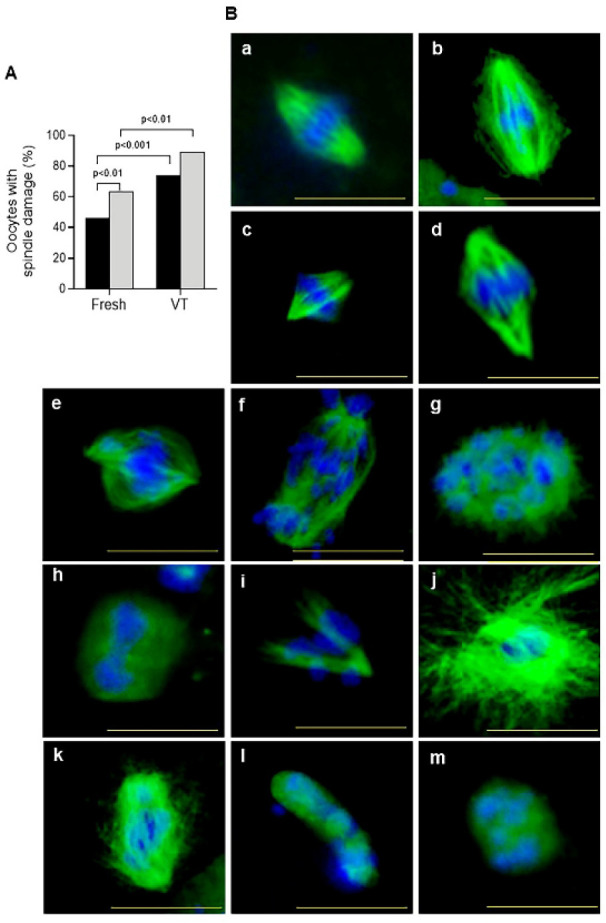
Effect of high-fat diet on meiotic spindle organization in MII oocytes derived from IVM of fresh and vitrified-thawed GV oocytes. **(A)** Percentage of MII oocytes with spindle damage (fresh-control: n=26, obese: n=25; VT-control: n=22, obese: n=18); **(B)** Representative images from (**a, b)** oocytes with normal spindle organization, and **(c-m)** oocytes with abnormal spindle organization (400x magnification). The scale bar indicates 20 µm. Control: 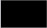 Obese: 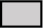

Feeding mice with HFD resulted in oocytes with significantly higher spindle defects (*p*<0.01) compared to oocytes from mice fed with chow diet. Vitrification and thawing of GV oocytes further increased spindle defects significantly in both control (p<0.001) and obese (p<0.01) groups. Additionally, a considerably higher percentage of spindle defects was observed in vitrified-thawed oocytes from the obese group compared to those from the control group (Figure 4, A and B).

### Effect of HFD-induced obesity on ER stress in GV oocytes

**Figure 5:**
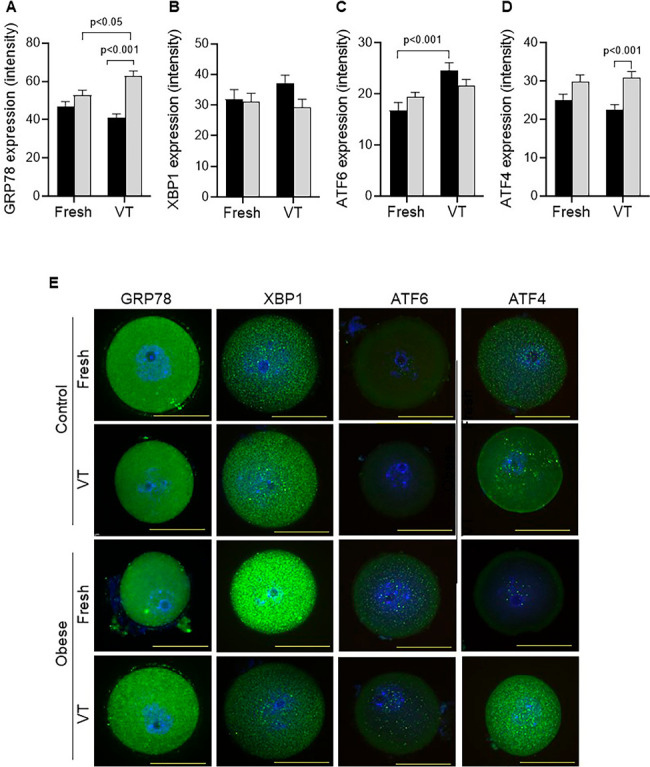
Effect of a high-fat diet and vitrification on the expression of ER stress markers in GV oocytes. **(A)** Expression of GRP78 in oocytes (fresh-control: n=30 and obese: n=30, VT-control: n=30 and obese: n=29); **(B)** Expression of XBP1 in oocytes (fresh-control: n=30 and obese: n=33, VT-control: n=30 and obese: n=30); **(C)** Expression of ATF6 in oocytes (fresh-control: n=30 and obese: n=32, VT-control: n=30 and obese: n=32); **(D)** Expression of ATF4 in oocytes (fresh-control: n=30 and obese: n=28, VT-control: n=30 and obese: n=33); **(E)** Representative images showing GRP78, XBP1, ATF6, and ATF4 expression in GV oocytes; (400x magnification). The scale bar represents 20 µm. The data are presented as mean ± SEM. Control: 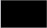 Obese: 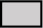

The expression of GRP78, XBP1, ATF6 and ATF4 in fresh and vitrified-thawed GV oocytes from the control and obese groups was assessed using immunofluorescence (Figure 5, A, B, C, D, and E). In fresh GV oocytes, there was no significant difference in GRP78 expression between the obese group and the control. However, in vitrified-thawed oocytes, the expression was significantly higher in the obese group (*p*<0.001) compared to the control (Figure 5, A and E). No significant difference in XBP1 expression was observed between control and obese groups in both fresh and vitrified-thawed oocytes (Figure 5, B and E). The expression of ATF6 did not differ between fresh oocytes of control and obese groups. In the control group, ATF6 expression was significantly increased (p<0.001) in vitrified-thawed oocytes compared to fresh ones (Figure 5, C and E). No change in ATF6 expression was observed between fresh and vitrified-thawed oocytes in the obese group. The expression of ATF4 was higher in the fresh and vitrified-thawed oocytes of the obese group compared to those of the control group, respectively (Figure 5, D and E), though the difference was not statistically significant. However, a significant increase in ATF4 expression was observed in vitrified-thawed oocytes of the obese group (*p*<0.001) compared to vitrified-thawed oocytes of the control group.

## DISCUSSION

In this study, we report for the first time that maternal obesity significantly affects the cytoplasmic reorganization of GV oocytes subjected to vitrification and thawing, even though it does not influence post-thaw survival. A decrease in the nuclear maturation rate *in vitro*, abnormal distribution of key organelles such as mitochondria and endoplasmic reticulum, disrupted spindle organization, elevated intracellular ROS levels, and ER stress were observed in vitrified-thawed oocytes of obese mice.

Earlier studies have shown that oocyte survival is largely influenced by the intrinsic quality of the oocyte and its susceptibility to damage from freeze-thaw processes (Fabbri *et al*., 2001). GV oocytes are very sensitive to cryoinjury because of their high water content, large size, and complex intracellular organization (Brambillasca *et al*., 2013). Our results show that the survival rate of GV oocytes after vitrification and thawing is 89%, which is similar to the 86% survival rate reported by Suo *et al*. (2009) in GV oocytes following vitrification and thawing.

Oocytes from mice fed with HFD are known to have elevated lipid content, higher ROS levels, and defective mitochondrial membrane potential (Marei *et al*., 2020; Rao *et al*., 2020). Elevated lipid levels lead to an increase in non-esterified fatty acids (NEFA), which are highly susceptible to lipid peroxidation (Meulders *et al*., 2025) and can cause significant damage to intracellular organelles such as the ER and mitochondria (Wu *et al*., 2010). Among the free fatty acids (FFAs), specifically saturated NEFA can have detrimental effects on oocyte quality and embryo development (Shi & Sirard, 2022). Supplementing NEFA during IVM of cumulus-oocyte complexes results in increased ER stress in oocytes, indicating their detrimental effects (Khatun *et al*., 2020; Kunnath *et al*., 2024). FFAs stored in lipid droplets are typically transferred into mitochondria to undergo β-oxidation for energy production, while some remain in the cytosol and either undergo peroxidation, producing ROS, or cause ER stress (Shi & Sirard, 2022). The increased oxidative stress observed in the oocytes of obese mice in this study confirms this.

Oocytes from obese mice are known to have poor cytoplasmic quality and developmental competence (Boots *et al*., 2016; Rao *et al*., 2020). Consistent with previous findings that exposure of oocytes to extreme low temperatures and high concentrations of DMSO during vitrification and thawing processes can induce ROS production in oocytes (Cao *et al*., 2022; Tatone *et al*., 2010), we observed a significant increase in ROS levels following vitrification in both control and obese groups. Interestingly, while vitrification independently elevated intracellular ROS levels, obesity did not appear to further exacerbate ROS production after vitrification and thawing. However, the *in vitro* maturation potential of oocytes was poor in vitrified-thawed oocytes from obese mice. Previous studies have reported a significant reduction in maturation rates of vitrified-thawed GV oocytes due to decreased intracellular cAMP levels and impaired activation of the metaphase-promoting factor (Ezoe *et al*., 2015; Shahedi *et al*., 2013; Zhao *et al*., 2011). Further, these maturation impairments have been linked to disruptions in the function of critical cellular structures, including mitochondria (Babayev & Seli, 2015; Lowther *et al*., 2009; Nohales-Córcoles *et al*., 2016; Zhou *et al*., 2024), the ER (Rho *et al*., 2002), spindle apparatus (Huang *et al*., 2008; Khalili *et al*., 2017), and cytoskeleton (Jia *et al*., 2018). A study conducted by Manipalviratn *et al*. (2008) found that vitrification and thawing of immature oocytes significantly reduced ATP concentration compared to fresh oocytes. In our study, we observed that the maturation rate further decreases in oocytes from obese mice following vitrification and thawing, which aligns with earlier reports by Jia *et al*. (2018). The decrease in the nuclear maturation potential of oocytes from obese mice could be due to defective mitochondrial function (Babayev & Seli, 2015). Mitochondrial dysfunction can compromise oocyte quality and reduce developmental potential (Hou *et al*., 2016; Wu *et al*., 2010). Earlier studies have demonstrated that the vitrification process results in the loss of mitochondrial membrane potential, decreased ATP synthesis and levels, impaired mitochondrial organization, and altered calcium oscillations in oocytes (Cao *et al*., 2009; Kasapi *et al*., 2017; Manipalviratn *et al*., 2008; Sun *et al*., 2004; Zhao *et al*., 2011). The impairment in mitochondrial localization observed in vitrified-thawed oocytes in this study may be due to damage to the microtubule network and disruptions in cytoskeletal dynamics (Zhu *et al*., 2024). Furthermore, mitochondrial aggregation could result from leaky mitochondrial membranes following vitrification and thawing. Additionally, increased ROS production in oocytes due to vitrification impairs the respiratory chain, leading to a decrease in mitochondrial DNA copy number (Amoushahi *et al*., 2017). Xu *et al*. (2021) observed that vitrification increases the co-localization of mitochondria and lysosomes in oocytes due to activation of mitophagy, indicating increased fusion between damaged mitochondria and lysosomes.

Oocyte maturation, fertilization, and early embryonic development depend on the functional integrity of the ER (Chen *et al*., 2024). Previous studies have demonstrated that the ER experiences significant stress during vitrification due to altered intracellular calcium (Ca^2+^) homeostasis and disrupted ultrastructure of the smooth endoplasmic reticulum (Lowther *et al*., 2009; Mo *et al*., 2025). Exposure to high concentrations of cryoprotectants and low temperatures alters ER homeostasis, impairs proper protein folding, and causes misfolded proteins to accumulate in the ER lumen, leading to ER stress. A key indicator of ER stress is the upregulation of chaperone proteins such as GRP78, XBP1, and C/EBP homologous protein (CHOP), which reflect disturbed Ca^2+^ regulation (Mo *et al*., 2025). The vitrification process induces ER stress and activates the unfolded protein response (UPR) signaling pathway (Zhao *et al*., 2015). Significantly higher levels of GRP78, XBP1, ATF4, and ATF6 proteins were observed in vitrified-warmed mouse oocytes (Barrera *et al*., 2018). Furthermore, our study’s results agree with previous findings, demonstrating a strong association between vitrification-induced ER stress and impairments in oocyte quality and maturation potential (Jia *et al*., 2024; Lowther *et al*., 2009).

Proper organization and function of the oocyte cytoskeleton are essential for normal chromosome segregation, spindle rotation, cytokinesis, pronuclei/nuclei formation, and syngamy (Bennabi *et al*., 2016; Londoño-Vásquez *et al*., 2022; Maro *et al*., 1986; Mogessie *et al*., 2018). Disruption of the cytoskeletal microtubular network within the oocyte can lead to spindle displacement and changes in chromosomal segregation, resulting in aneuploid oocytes or embryos (Dunkley & Mogessie, 2023; Nikalayevich *et al*., 2024). Microtubules are highly sensitive to temperature fluctuations and cryoprotectants, which can cause depolymerization and disorganization of the spindle during freeze-thaw processes. Additionally, cryoprotectants such as DMSO have been shown to impair spindle polymerization (Gomes *et al*., 2012). The vitrification medium contains high concentrations of penetrating cryoprotectants, which may negatively affect microtubule organization. DMSO in vitrification solutions, depending on exposure duration and concentration, can adversely impact oocyte and embryo quality. Actin distribution, spindle migration, asymmetric division, and cytokinesis are impaired during oocyte meiotic maturation, leading to abnormal cell division in oocytes exposed to DMSO concentrations ranging from 2% to 4%, with maturation blocked at 6%. Besides DMSO, permeable cryoprotectants such as ethylene glycol and glycerol also induce failure of asymmetric division (Zhou *et al*., 2014).

Obesity induces alteration in actin filament dynamics causing aberrant spindle morphology and misaligned chromosomes (Igosheva *et al*., 2010). The osmotic shock during equilibration may cause oocytes to shrink and deform, potentially damaging the cytoskeleton and microfilaments. Further, cytoplasmic lipid droplets in ooplasm are prone to cryoinjury (Mogas *et al*., 2024; Schallmoser *et al*., 2024). This suggests that obesity exacerbates the vulnerability of the spindle apparatus to cryopreservation-induced stress. These findings align with earlier research indicating that spindle defects are more prevalent in oocytes from obese individuals, likely due to cytoskeletal alterations and energy deficits stemming from mitochondrial dysfunction (Rao *et al*., 2020). The further increase in spindle abnormalities after vitrification in both control and obese groups indicates that cryopreservation exerts additional stress to the oocytes, with a more significant impact on the obese group. These findings have important implications for ART, particularly for obese women undergoing oocyte vitrification. The poorer oocyte quality and higher spindle defects observed in the obese group suggest that strategies to enhance organelle function, such as antioxidant supplements or metabolic optimization, might improve oocyte quality during vitrification and thus improve ART outcomes. Further research into strategies to mitigate these effects could enhance ART success rates in obese patients.

Overall, the results of the present study indicate that maternal obesity does not have significant adverse effects on the cryosurvival of oocytes; however, it negatively impacts oocyte quality, with important implications for ART in obese women. We acknowledge that this study has some limitations, especially regarding the understanding of embryonic outcomes of oocytes. The obese mice model used in this study may not fully account for the multifactorial nature of human obesity, which includes genetic, environmental, and lifestyle factors, since HFD-induced obesity in mice mainly reflects weight gain from caloric excess. Using the HFD-induced mice model could limit the broader relevance of our findings, as obesity in women with varying degrees may differently impact reproductive outcomes. Employing more physiologically relevant obesity models would improve the understanding of how maternal obesity affects cryopreservation outcomes and subsequent pregnancy success in ART.
